# Assessment of Ammonia Emissions and Greenhouse Gases in Dairy Cattle Facilities: A Bibliometric Analysis

**DOI:** 10.3390/ani14121721

**Published:** 2024-06-07

**Authors:** Patricia Ferreira Ponciano Ferraz, Gabriel Araújo e Silva Ferraz, Jacqueline Cardoso Ferreira, João Victor Aguiar, Lucas Santos Santana, Tomas Norton

**Affiliations:** 1 Department of Agricultural Engineering, School of Engineering, Federal University of Lavras (UFLA), Lavras 37200-900, Brazil; gabriel.ferraz@ufla.br (G.A.e.S.F.); jacardosof@gmail.com (J.C.F.); 2 Department of Animal Science, Faculty of Animal Science and Veterinary Medicine, Federal University of Lavras (UFLA), Lavras 37200-900, Brazil; joao.aguiar@estudante.ufla.br; 3 Department of Agricultural and Environmental Engineering, Federal University of the Jequitinhonha and Mucuri Valleys (UFVJM), Unaí 38610-000, Brazil; lucas.unemat@hotmail.com; 4 Department of Biosystems, KU Leuven, Kasteelpark Arenberg 30, B-3001 Leuven, Belgium; tomas.norton@kuleuven.be

**Keywords:** air pollution, dairy cow, environmental sustainability, systematic review

## Abstract

**Simple Summary:**

Dairy farming is a significant source of pollutant gas emissions, contributing to climate change and air quality degradation. Understanding these emissions in milk production promotes productive efficiency and sustainable resource use. The aim of this paper is to provide a comprehensive analysis of ammonia and greenhouse gas emissions in dairy farming, utilizing bibliometric analysis methods. Articles featuring experimental data collection were selected, exclusively in English. The databases considered were Scopus and Web of Science. The analyses were conducted using Bibliometrix version 4.0.0 and VOSviewer version 1.6.20 tools to create knowledge maps visualizing information on research countries, institutions, author collaborations, and keyword networks. The search yielded 187 articles related to major pollutant gases such as methane, ammonia, carbon dioxide, nitrous oxide, and hydrogen sulfide. This study provided valuable insights into gas emission sources, air quality, environmental sustainability, and animal welfare, as well as identifying gaps and critical points in research on the topic. Ammonia and greenhouse gas emissions in dairy farming are crucial for climate change mitigation and sustainable agriculture. This research can contribute to academic interests while enhancing agricultural practices and informing environmental policies. The relevance of this research will persist with the growing demand for sustainable practices, as more researchers and organizations recognize the importance of gas emissions and mitigation research.

**Abstract:**

A deeper understanding of gas emissions in milk production is crucial for promoting productive efficiency, sustainable resource use, and animal welfare. This paper aims to analyze ammonia and greenhouse gas emissions in dairy farming using bibliometric methods. A total of 187 English-language articles with experimental data from the Scopus and Web of Science databases (January 1987 to April 2024) were reviewed. Publications notably increased from 1997, with the highest number of papers published in 2022. Research mainly focuses on ammonia and methane emissions, including quantification, volatilization, and mitigation strategies. Other gases like carbon dioxide, nitrous oxide, and hydrogen sulfide were also studied. Key institutions include the University of California–Davis and Aarhus University. Bibliometric analysis revealed research evolution, identifying trends, gaps, and future research opportunities. This bibliometric analysis offers insights into emissions, air quality, sustainability, and animal welfare in dairy farming, highlighting areas for innovative mitigation strategies to enhance production sustainability. This research contributes to academia, enhancing agricultural practices, and informing environmental policies. It is possible to conclude that this research is a valuable tool for understanding the evolution of research on gas emissions in dairy cattle facilities, providing guidance for future studies and interventions to promote more sustainable production.

## 1. Introduction

The production of milk is significant globally, playing a fundamental role in reducing poverty, enhancing food security, and providing nutrition for billions of people worldwide. Additionally, it has contributed to a 40% increase in economic value over the past decade [1].

Nevertheless, the escalating global population has driven an increased demand for food resources, including milk. Consequently, the increase in milk production has led to heightened levels of manure production, resulting in notable emissions of greenhouse gases (GHGs) and ammonia (NH_3_), posing significant threats to the climate and air quality [2]. The production of milk and gas emissions may have a complex relationship. While increased milk production often requires higher feed consumption, leading to greater gas emissions, strategies such as enhancing feed quality and digestibility can improve productivity while simultaneously reducing gas production per unit of milk.

The major challenges facing the dairy industry today include environmental concerns that profoundly impact its sustainability, along with social and economic considerations [3]. Assessing the overall efficiency of the dairy industry involves correlating GHG and NH_3_ emissions with milk production [4]. This evaluation emphasizes the importance of mitigating resource consumption in production and highlights the potential for improving productivity efficiency to reduce environmental impacts.

Dairy production is environmentally impacted by various sources, including GHG emissions originating from enteric fermentation, manure storage, and other agricultural practices. Additionally, the use of water resources for feed production impacts water quality due to nitrogen and phosphorus excretion. Furthermore, land requirements for feed production contribute to the overall environmental footprint [5].

In the context of gases emissions from dairy farming, especially in intensive animal production, significant emissions occur, including ammonia (NH_3_), methane (CH_4_), carbon dioxide (CO_2_), nitrous oxide (N_2_O), and hydrogen sulfide (H_2_S) [6,7,8].

NH_3_ constitutes the most-emitted gas from dairy production, while other air pollutants include reactive nitrogen species (e.g., nitrogen oxides and NO_2_), odor emissions (e.g., organic acids), and sulfur compound gases (e.g., H_2_S) [7].

According to Schmithausen et al. [9], gases such as CH_4_, CO_2_, and N_2_O are among the most relevant GHGs and are considered significant contributors to global climate change. These gases are primarily produced by enteric fermentation, feed production, diet management, manure, and total product output.

Assessing emission rates poses a complex challenge due to numerous factors impacting emissions, such as the variety and quantity of animals, nutrient inputs and feeding practices, housing conditions, manure-handling methods, and environmental factors [10].

One approach to comprehending the nuances of research on NH_3_ and GHG emissions in dairy production systems is to apply bibliometric methodology. According to Chistov et al. [11] and Marin et al. [12], bibliometric analyses employ mathematical and statistical techniques to explore and measure metadata to organize, analyze, and examine bibliographic material. According to Donthu et al. [13], the use of bibliometric techniques for analyzing quantitative data allows for the construction of indicators regarding the dynamics and evolution of scientific and technological information. These indicators provide a deeper understanding of the scientific and technological landscape, highlighting trends and changes over time (Sarkar et al. [14]).

Bibliometric analysis helps researchers explore, organize, and analyze large volumes of information, uncovering hidden patterns that can be used in the decision-making process [15]. In summary, the insights gained from these analyses shed light on trends in GHG research and highlights research practices and scientific production [16].

Based on this, a bibliometric analysis of gas emissions in dairy production systems can offer valuable insights into the complex interactions between emissions and the dairy industry. This study will enhance our understanding of these dynamics and provide a more detailed and accurate overview of the research landscape.

This paper aims to conduct a comprehensive bibliometric analysis to elucidate the evolution of scientific research on NH_3_ and GHG emissions in dairy production systems. The objective is to provide a differentiated view of existing research, outlining a path for future investigations, while simultaneously deepening the quantitative exploration of this field based on an extensive academic foundation.

## 2. Materials and Methods

**Figure 1:**
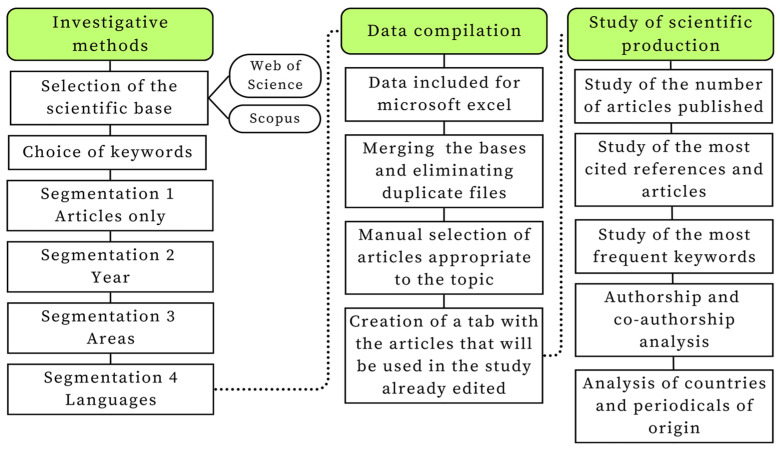
Bibliometric analysis processes. Adapted from Santana et al. [16].

We used bibliometric analysis as a quantitative method to track the progress of scientific production related to gas emissions in dairy farming, and the framework for this study is shown in Figure 1.

The adopted sequence of activities in this biometric analysis includes data retrieval, pre-processing, network extraction, normalization, mapping, and analysis [17].

### 2.1. Scientific Database Selection

The Scopus and Web of Science databases were chosen for their global recognition and significance in scientific literature [18]. Searching various scientific databases is vital for accurately interpreting and using bibliometric indicators in scientific research evaluation. Bibliometric analysis on multiple topics typically uses at least one of these databases [19].

We conducted an extensive search of journal articles covering research on ammonia and GHG emissions in dairy production systems. The study selected peer-reviewed scientific articles published between January 1987 and April 2024. Preference was given to articles that provided original research findings and comprehensive author and affiliation information. The selection criteria focused exclusively on scientific articles, particularly those conducting experimental analyses in real cow facilities or controlled environments such as chambers, wind tunnels, and labs. Furthermore, to ensure the universality and authenticity of the acquired data, publications in languages other than English were excluded [20].

### 2.2. Data Research Criteria

Seeking to conduct a comprehensive bibliometric analysis the relevant keywords for this research were defined based on animal species AND type of housing, AND type of gases. Different searches were carried out to define the keywords to identify the ones most used by authors in the field of study as suggested by Marin et al. [12]. Articles were exclusively considered in the research if they contained the combination of the specified keywords in the title, abstract or keywords section (and acronyms).

Brackets were used to ensure an exact match for individual words. Boolean operator OR was applied to guarantee that each search result contained at least one of the designed terms: (“dairy cattle” OR “dairy cow” OR “dairy cows”) AND (“building” OR “buildings” OR “barn” OR “barns” OR “shed” OR “sheds” OR “housing” OR “house” OR “houses” OR “stall” OR “stalls” OR “compost bedded pack” OR “compost-bedded” OR “compost bedded” OR “compost bedding” OR “feedlot” OR “confinement”) AND (“greenhouse gas” OR “gas” OR “gases” OR “carbon dioxide” OR “nitrous oxide” OR “emission” OR “emissions” OR “methane” OR “ammonia”).

### 2.3. Selection Procedures and Data Organization

First, duplicate articles from the Scopus and Web of Science research databases were removed manually. In the second stage, conceptual works without experimental analyses in real cow facilities or in controlled environments were excluded. This study specifically focused on articles that demonstrated the collection of experimental data on atmospheric gas emissions. Theoretical articles or those containing data on enteric gases were excluded from the analysis. From a total of 636 articles, the process excluded 449 articles unsuitable for research, resulting in 187 articles that were the focus of the study.

Subsequently, the data from each selected article were organized in an electronic spreadsheet and imported into bibliographic analysis software.

### 2.4. Data Analysis

For the analysis, RStudio^®^ software version 4.4.0 and the Bibliometrix (version) 4.0.0 data package were used, in order to generate graphs of responses to the searches applied.

The data entry was related to the following information: Abstract (AB), Affiliation (AF), Author (AU), Corresponding Author (CA), DOI (DI), Document Type (DT), Journal (JO), Keywords (KE), Keywords Plus (KP), Language (LA), Publication Year (PY), Title (TI), Total Citation (TC), Cited References (CR), and Science Categories (SC).

To obtain analysis related to co-authorship, co-occurrence, citations, graphic coupling, and co-citations, VOSviewer version 1.6.20 was used. Using this software, analyses of authors, organizations, countries, keywords, documents, sources, and references were also generated.

**Figure 2:**
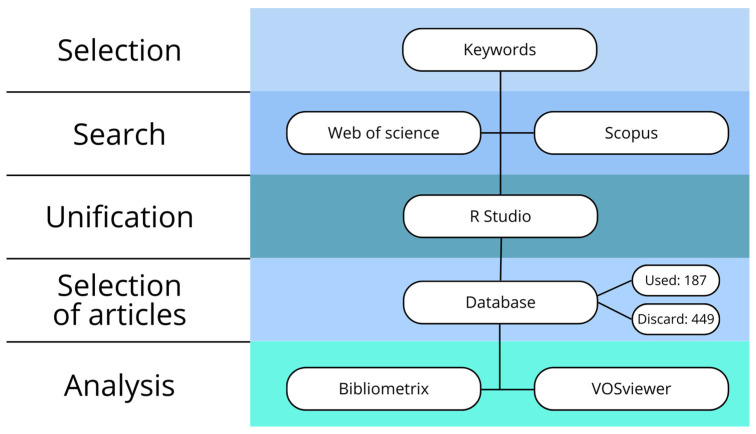
Processes adopted for bibliometric analysis.

The file selection processes can be described as shown in Figure 2.

### 2.5. Data Interpretation

In this analysis, the 187 selected articles were categorized based on the type of housing system where the experiments were conducted (free stall, tie stall, loose housing, compost barn, feedlot, open lots), or if the experiments were carried out in a controlled environment such as labs conditions, chambers, wind tunnels and labs. Additionally, categorization was performed concerning the main gases discussed in the articles, including methane (CH_4_), ammonia (NH_3_), carbon dioxide (CO_2_), nitrous oxide (N_2_O), and hydrogen sulfide (H_2_S).

From the dataset submitted for bibliometric analysis, cluster maps were generated, depicted by colors, for author co-citations, identifying collaboration networks, areas of common interest, and academic influence [21]. The most frequently used keywords were also examined, determining the trend of their usage over time. Furthermore, we analyzed the annual evolution of publications, the principal researchers, the most influential countries on the topic, prominent journals, academic institutions involved, and the main areas of knowledge, as well as future research directions.

The objective of this analysis is to ease the interpretation of the selected articles and comprehend the evolution of research concerning the types of facilities. Additionally, it aims to explore the research interest in relation to the types of gases analyzed over the studied years.

## 3. Results and Discussion

### 3.1. Annual Scientific Publications on Gases in Dairy Cattle Farming

**Figure 3:**
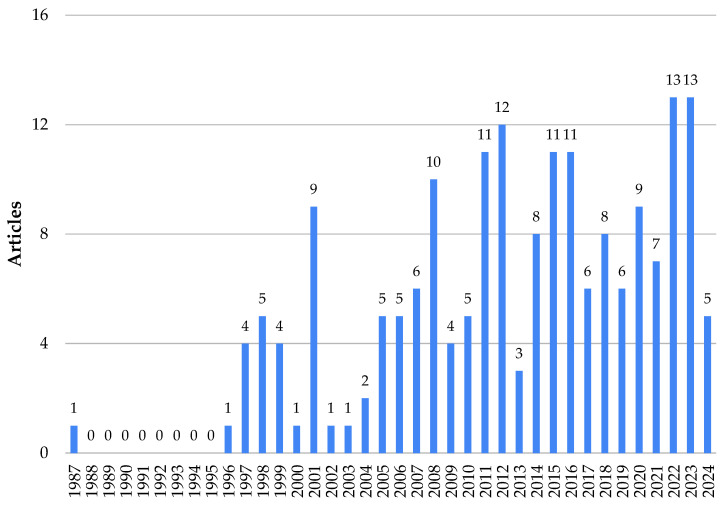
Number of publications per year concerning gas emissions in milk production from 1987 to April 2024.

The analysis of Figure 3 reveals that studies on gas emissions began to emerge in 1987, with the publication of only one article.

For centuries, dairy production has been a crucial component of agricultural and food systems worldwide. Livestock manure emits considerable amounts of NH_3_ and GHGs, inducing climate change and air pollution [2]. Understanding gas emissions in dairy cattle farming has been a topic of increasing interest in recent decades, reflecting the recognition of the relationship between milk production and environmental challenges.

The first article assessed NH_3_ emissions measured from livestock excreta and fertilizers. Based on the findings of this study, combined with information from other published results, an NH_3_ emission inventory was calculated for pasture and livestock production systems across the United Kingdom [22].

In the period from 1988 to 1995, no articles related to gas emissions in dairy production were published. After that, the evolution of publications on this topic reveals a trajectory marked by significant advances in understanding the environmental impacts associated with gas emissions in livestock farming. Since the first investigations into gas emissions associated with dairy farming, research has evolved to address not only the quantitative aspects, but also to explore innovative and sustainable mitigation strategies. Growing global awareness about climate change and the pressing need to adopt more sustainable agricultural practices have highlighted the importance of scientific investigations in this specific issue.

During this period, there was a heightened global concern among nations regarding the emission of greenhouse gases and their potential impact on climate change and global warming. This period coincided with significant global initiatives, including the United Nations Framework Convention on Climate Change (UNFCCC) in 1992. Serving as the primary forum for international cooperation on GHG-induced climate change, its objective was to “stabilize greenhouse gas concentrations in the atmosphere at a level that will prevent dangerous human interference with the climate system, in a time frame that allows ecosystems to adapt naturally and enables sustainable development” [23].

From 1996 to 2006, little research were carried out considering the emission of gases from dairy farming, of which the majority deepened in NH_3_ emissions from manure. However, in 2006, the Intergovernmental Panel on Climate Change (IPCC) [24] developed methodological guidelines for national GHG inventories, special reports and technical documents. The IPCC developed three working groups: GT I—The science of climate change; GT II—Impacts, adaptation and vulnerability and GT III—Climate change mitigation; used according to the types of data available [25].

It is interesting to consider that the majority of climate policy legislation has been established since the early 2000s, with the most significant activity observed between 2007 and 2012 [26,27]. This timeframe coincides with the implementation of the Kyoto Protocol—one of several international factors that have stimulated the development of climate policies [26]. Adopted in 1997, the Kyoto Protocol came into effect in 2005 and underwent its first phase of binding constraints from 2008 to 2012 [28].

In this period, there was an increase in the number of publications, reflecting growing concerns about gas emission, the effects of global warming and climate change. Research efforts focused on exploring strategies to mitigate gas emissions in dairy farming, such as modifying feed formulations to adjust forage/concentrate ratios, varying corn kernel size, reducing crude protein content, and incorporating supplements [29,30,31,32,33]. Sustainable waste management practices were also discussed in these documents, including the use of slatted floors to improve waste drainage, the adoption of bedding with organic materials to help reduce emissions [34,35,36], and controlling the frequency of litter changing and storage, as well as separating liquid manure, as important additional measures [37,38].

In 2015, during the United Nations (UN) climate conference in Paris, 180 countries agreed to further reduce greenhouse gas emissions to meet the “below 2 °C target” by the end of the 21st century [39]. This period was a timeframe of stronger growth with higher numbers of articles per year. This agreement may stimulate an increase in research related to the mitigation of gas emissions. As a result, there has been a surge in efforts to develop innovative strategies and technologies aimed at measuring and mitigating emissions from various sectors, including the dairy cattle industry.

From 2020 onwards, monitoring and quantification of gases could be carried out in real time through measurement systems integrated into unmanned aerial vehicles (UAVs) [40]. Non-invasive measurement practices, such as gas measurement by mass balance, or the use of infrared equipment, contribute to maintaining animal welfare [41,42,43]. Furthermore, the estimate of other gases can be obtained through the carbon dioxide balance method, which is useful for evaluating the effectiveness of management and ventilation practices in reducing greenhouse gas emissions in livestock facilities [44].

The rise in publication numbers since 2022 implies heightened research activity, likely caused by increased global awareness of gas emission, a growing focus on sustainable agricultural practices, and the imperative to confront climate-related challenges. Additionally, the global impact of NH_3_ and greenhouse gas emissions research underscores its capacity to inform policy decisions and influence agricultural practices. This surge in research output regarding gas emissions from dairy production in recent years has the potential to inform policy, steer sustainable agricultural practices, and enhance public awareness of the livestock sector’s role in climate change. This surge denotes the dynamic nature of the field studies and its practical implications [45].

### 3.2. Main Gases Studied in Scientific Articles over the Years

**Figure 4:**
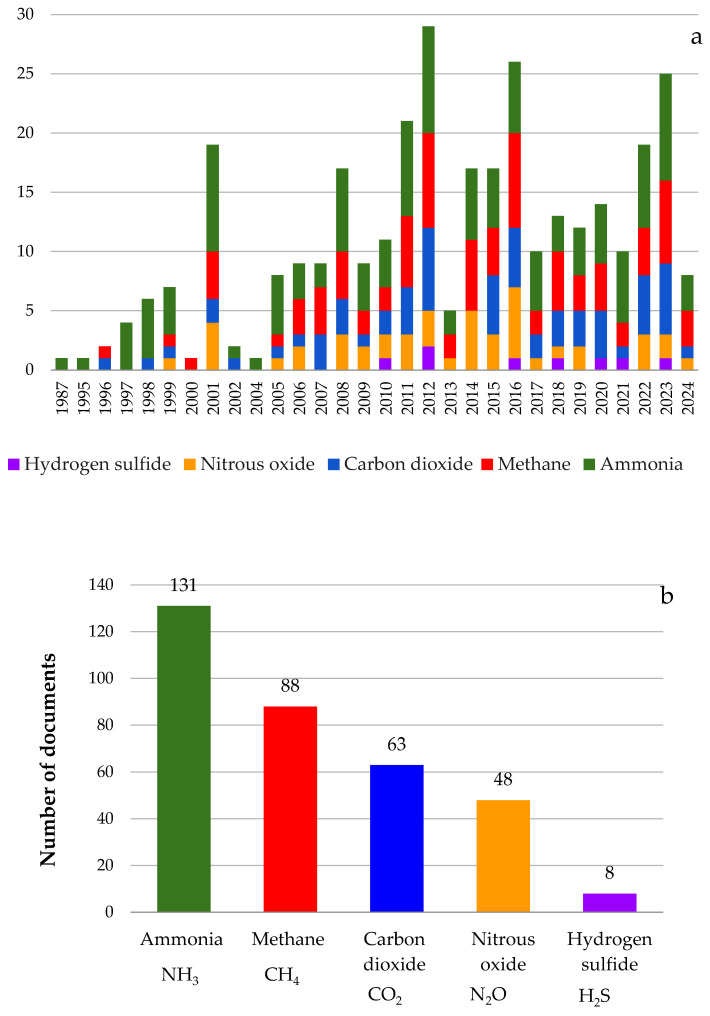
Gases investigated in articles from January 1987 to April 2024 (**a**), frequency of gases per documents during the evaluated period (**b**).

In Figure 4, it is possible to observe the types of gases investigated in each scientific article within the context of dairy farming over the years.

Figure 4a,b elucidates the evolving of scientific research highlighting the types of gases examined between January 1987 to April 2024, emphasizing the frequent interest in NH_3_ and CH_4_. Dairy barns, particularly in the context of intensified dairy production, emerge as significant sources of NH_3_ and CH_4_ emissions [46,47]. The emissions of these gases stemming from livestock production represent a global environmental concern [48].

Research conducted during this period predominantly centers on NH_3_ and CH_4_ emissions, encompassing their quantification and volatilization processes, aimed at identifying effective mitigation strategies. NH_3_ is generated through the microbial breakdown of organic nitrogen compounds present in manure. Nitrogen exists in the form of unabsorbed nutrients in animal feces and as either urea (in mammals) or uric acid (in poultry) in urine [49]. The main source of CH_4_ emissions in livestock farming comes from the microbial fermentation of cellulosic feed material in the rumen or, to a lesser extent, in the intestine, while a minor fraction is generated during manure decomposition [8,50]. These gases contribute to global warming, eutrophication of ecosystems, and airborne particulate matter pollution [49,50,51,52]. Accurate quantification of these emissions could aid in developing effective mitigation strategies and emission inventories.

After NH_3_ and CH_4_, the most-researched gases over the years evaluated were carbon dioxide (CO_2_), nitrous oxide (N_2_O), and hydrogen sulfide (H_2_S), respectively (Figure 4b).

Livestock respiration contributes significant amounts of CO_2_ [53]. CO_2_ is a natural component of the air and part of the animal respiration process [41]. High concentrations of CO_2_ can cause irritation in the respiratory tract of animals, compromising the well-being and sustainability of the dairy industry [54].

N_2_O is a byproduct of enteric fermentation and is one of the responsible for the deposition of nitrogen in the ecosystem [55]. According to Amon et al. [6], the estimates of N_2_O emissions from manure management are based on the assumption that a certain percentage of nitrogen excreted by domestic livestock is released as N_2_O during manure storage. There are many uncertainties regarding the factors that can influence the emission of this gas, but it is known that N_2_O emissions during storage vary based on the nitrogen and carbon levels in manure, the accumulation time, the treatment method employed, frequency and amount of substrate added to the bedding, animal type, and feeding system [55,56]. N_2_O stands as a potent GHG, with its emission in the ecosystem raising significant concerns due to its longevity as an atmospheric trace gas [57]. Its global warming potential, at 265 times that of CO_2_, combined with a lifespan exceeding 100 years, underscores its environmental impact [58].

H_2_S is produced as manure decomposes anaerobically, resulting from the mineralization of organic sulfur compounds as well as the reduction of oxidized inorganic sulfur compounds, such as sulfate, by sulfur-reducing bacteria [7,59]. H_2_S can be considered the most dangerous gas in dairy production, capable of inducing respiratory, ocular, and neurological diseases. Moreover, its presence poses a fatal risk to both animals and farm workers within livestock facilities [60]. Few articles measured H_2_S concentrations in dairy barns because the comprehensive estimation of total H_2_S emissions from dairy production remains challenging due to the absence of essential data required for accurate estimation [60]. This is due to the fact that H_2_S emissions are influenced by various factors, including manure production and storage, disturbance of manure, climatic conditions (particularly temperature, air humidity, and ventilation), feeding methods, ration composition, and the ambient configuration of dairy barns [60]. However, it is important to note that these factors may not always be readily available or easily quantifiable.

### 3.3. Impacts of Livestock Manure Emissions on Environmental Sustainability and Animal Welfare

Environmental issues are one of the major factors affecting the sustainability of the dairy industry, alongside social and economic factors [7]. According to Ribeiro-Filho et al. [61], gas emissions from livestock activities represent 10–12% of global emissions, with almost 30% coming from dairy cattle production systems. Therefore, the production of dairy products is intricately linked to environmental impacts such as global warming, land use changes, freshwater eutrophication, and water depletion [62,63]. Livestock manure is recognized as a significant source of GHG emissions and NH_3_, contributing to climate change and air pollution [2]. For this reason, the dairy industry is facing growing criticism for its contribution to global gases emissions [64].

In the current scenario of a climate emergency, characterized by rising greenhouse gas emissions and escalating extreme weather events [65], climate change poses a significant challenge. Climate change refers to the gradual alteration of temperature and weather patterns due to the accumulation of greenhouse gases in the atmosphere [64].

Dairy production is associated with the emission of various atmospheric pollutants and the primary sources of GHG emissions from dairy production systems are CO_2_, CH_4_, and N_2_O, which are primarily produced through enteric fermentation, feed production, manure production, and management processes [66]. These three gases contribute negatively to the greenhouse effect, soil pollution, and aquatic pollution [67].

NH_3_ is the primary gas emitted in dairy systems, and it is recognized for its toxicity and contribution to environmental issues such as atmospheric acidification and soil and water eutrophication [68,69]. Additionally, NH_3_ reacts with other compounds, such as sulfur and nitrogen oxides, contributing to global warming through the formation of N_2_O, which has a warming potential 273 times greater than CO_2_ [70].

Research by Guzmán-Luna et al. [63] highlights a clear interaction between the dairy sector and climate change. While the dairy sector significantly contributes to greenhouse gas emissions, it is also directly affected by these changes. According to IPCC [71], emissions from the dairy sector can contribute to climate change through processes such as temperature variation, sea level rise, ocean warming and acidification, melting sea ice, and extreme weather events (e.g., heat waves, droughts, heavy rainfall), as well as changes in ecosystems. Moreover, Calzadilla et al. [72] and Guzmán-Luna et al. [63] suggest that extreme weather events are becoming more frequent, posing challenges to crop productivity for animal feed and land distribution. This extreme weather events may lead to increased competition for suitable land and potential changes in global crop trade patterns. Consequently, regions vulnerable to significant temperature increases may experience adverse effects on dairy cattle, including increased heat stress, which negatively impacts their health, milk production, and fertility [73]. Additionally, rising temperatures pose risks to dairy product safety, increasing the likelihood of food spoilage and microbial contamination [74]. While the full extent of these effects remains uncertain, it is evident that they pose multifaceted threats to the dairy sector, influenced by geographical location [63].

Despite the increasing awareness of environmental challenges, there persists a knowledge gap regarding GHG and NH_3_ emissions from livestock manure, underscoring the necessity for additional research in this domain. Moreover, exploring the impacts of livestock manure emissions on environmental sustainability and animal welfare through research and publications is crucial. Understanding the intricate relationships between these emissions, environmental health, and animal well-being is essential for devising effective strategies to mitigate negative impacts and promote sustainable practices in the dairy industry. As such, further investigation and dissemination of findings in this field are vital for fostering a more environmentally conscious and ethically responsible approach to dairy production.

### 3.4. Gas Emissions by Type of Facility

**Figure 5:**
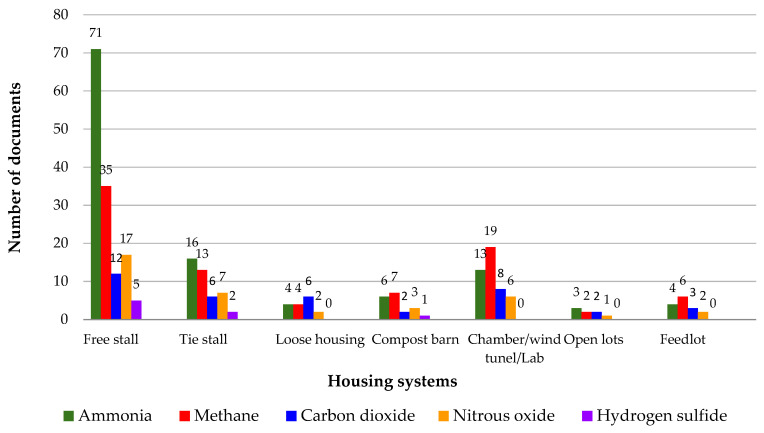
Number of published articles based on the types of gases and different types of housing systems for dairy cattle.

Gas emissions occur differently depending on the type of housing that houses the animals and the technological level adopted. It is important to identify which systems were studied throughout the analyzed period. According to Figure 5, it is possible to observe the number of published articles based on the types of gases and different types of housing systems for dairy cattle.

Dairy cattle are usually accommodated in housing systems with various modifications within each system based on factors such as bedding type, stall presence, and resting area dimensions, among others [75]. The type of the housing plays an important role in farm operations because it impacts various sustainability factors, including environmental considerations, the welfare of cattle, and economic aspects [76]. According to Bewley et al. [77], traditional housing systems for dairy cattle include conventional bedded-pack, tie stall, free stall, and compost bedded-pack barns. However, the early years of many peer-reviewed journals do not include information on dairy cattle housing.

The requirements and demands for dairy cow housing vary significantly among countries, leading to a diverse array of dairy housing systems.

Bewley et al. [77] conducted a study on the advancements in housing systems for dairy cattle across various systems over the past century. According to these authors, the tie stall system was regarded as the traditional type of dairy housing for many years. However, with the increase in herd sizes and changes in the dairy industry during the 20th century, this system lost some of its popularity among milk producers. While tie stalls continue to operate in many countries, free stall barns have become widespread since the 1970s [77]. Due to its popularity worldwide, this type of housing has emerged in terms of the quantity of scientific articles.

The compost barn confinement system is an alternative to the traditional loose housing system, where animals are free to walk within the barn [78]. The compost barn system has gained popularity in recent years due to its ability to enhance animal well-being and quality of life, thereby promoting productivity and longevity [17]. According to the same authors, as a relatively new housing system, it has attracted the attention of researchers and producers, resulting in a notable increase in scientific publications on compost barn housing in recent years.

According to Figure 5, it can be observed that many studies were conducted under laboratory conditions, chambers, or wind tunnels; all of them can be considered controlled environments. On a laboratory scale, the environment tends to be relatively constant, as they are typically carried out in a controlled room with stable ambient conditions. According to Kupper et al. [79], since many of these studies focused on comparing various techniques or systems, their primary aim was not necessarily to accurately represent emission rates under real-world conditions.

In this study, papers most found were conducted at real facilities on farm sites. This information was obtained from the description of the experimental setup provided in the papers. However, some research regarding NH_3_ and GHG emissions was carried out in laboratory-scale studies [80], dynamic flow-through chambers [81], emission chambers [46], wind tunnels [82], or any kind of controlled room. Moreover, the majority of the 187 papers analyzed utilized Holstein cows, with 160 articles specifically employing this breed, while 2 studies used Jersey cows, 2 utilized Fleckvieh, and 3 utilized crossbred cows. Some studies used more than one breed, with two studies using Holstein and Jersey breeds and one study using Holstein and crossbred breeds. Additionally, 23 articles did not specify the breeds used. Furthermore, in assessing gas emissions in dairy cattle housing, 12 evaluations were conducted using individual sensors, while 176 studies conducted evaluations of gas emissions collectively. Possible differences between Holstein and Jersey breed have called the attention of researchers to evaluate the main effects and interactions among dairy cow breed and dietary composition on GHG emissions measured from the cow, the manure and the field. However, per Uddin et al. [83], the assessed cow breeds (Holstein and 4 Jersey cows) and forage-neutral detergent fiber (FNDF) sources did not impact any of the measured CO_2_, CH_4_, or N_2_O fluxes or cumulative GHG emissions determined in their study.

In certain countries, there is an incentive for grazing due to the society preference to see cows in the landscape; open-lot grazing is believed to be beneficial for cow health and welfare [84]. Nevertheless, managing grazing can pose challenges for farmers with extensive herds, automated milking systems, and limited surrounding land [85]. Moreover, research and collecting gas emissions in open environments is challenging, which is why there are few publications on the subject.

### 3.5. Publications by Countries and Connection Networks

In the context of gas emissions from dairy farming, it is interesting to analyze and understand the distribution of global scientific production. This type of approach is interesting for evaluating the current state of research and identifying emerging trends, as well as identifying the countries that are most interested in the subject in question.

**Figure 6:**
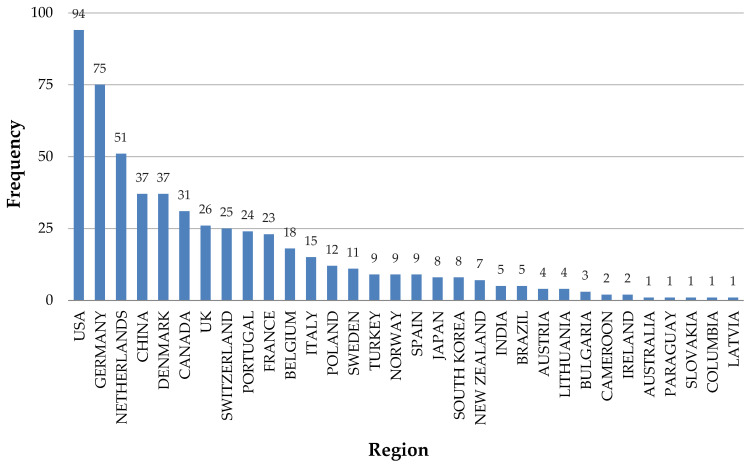
Number of articles published by countries between January 1987 and April 2024.

The global relevance of the topic, in the studied period can be expressed in terms of publication by country (Figure 6).

The United States topped the list of countries from which the documents were published during the years under study, with 94 published documents, followed by Germany (75) and the Netherlands (51), both from the European Union, and China with 37 publications. According to the PBL Netherlands Environmental Assessment Agency [86], China (30%), the US (15%), and the European Union (EU-28) (10%) collectively contribute to over 50% of total global greenhouse gas emissions.

In 2023, the European Union emerged as the primary global producer of cow milk, generating an impressive total of over 143 million metric tons. Following closely behind, the United States secured the second position, producing approximately 104 million metric tons of cow milk during the same period [87]. China produced approximately 42 million metric tons of cow’s milk, marking the highest volume in recent years [87]. Per capita milk consumption in China is much lower than the global level, and vigorous efforts are still needed to develop the dairy industry. Consequently, mitigation strategies for China’s dairy industry need to be extensively investigated [88].

The increase in productivity in dairy cow farming considers both animal numbers and the productivity of each individual. However, this increase in milk production over the past few years can only be achieved through the development of innovative research in dairy cattle farming [89].

Reducing GNG emissions from dairy production in the U.S. has become a political priority since the country rejoined the Paris Agreement in 2021 [90] and aligned with the U.S. dairy sector’s goal of achieving climate neutrality by 2050 [91].

During the Paris Agreement on climate change negotiations, the European Union (EU) committed to establishing a novel climate change mitigation target for 2030, pledging to reduce GHG emissions by a minimum of 40% compared to 1990 levels [92].

Due to rapid economic growth, China became the world’s largest GHG emitter, surpassing US emission levels in 2006 [93]. However, recently, China has increased awareness of the harmfulness of GHGs and taken measures to address them [88].

Based on the Figure 6, it is evident that countries such as the USA (94), Germany (74), the Netherlands (51), and China (37) exhibit remarkably high scientific production related to gas emissions in dairy cattle systems. These countries have a significant scientific output related to dairy farming and, consequently, to the associated gas emissions from this activity. In order to mitigate GHG emissions from dairy cows, it is essential to comprehend the distinct features of GHG emissions from these animals. Various factors such as breed, environmental conditions, dietary intake, and physiological status influence GHG emissions from dairy cows [88]. Achieving a thorough understanding of these emissions requires extensive research to establish a comprehensive database.

In Figure 6, it can be observed that the majority of published works during the analyzed period were from developed countries, with the United States and Europe standing out, despite many developing countries having much larger dairy outputs than these countries. This observation leads us to conclude that developing countries are not researching and quantifying gas emissions in dairy facilities. This may be due to a combination of factors such as lack of government funds, research incentive policies, appropriate infrastructure systems to make the measurements, and expertise; and environmental impacts being a lower research priority [94].

Over the last few decades, it is known that to quantify and to manage gas emissions and environmental impacts is a crucial part of dairy research. However, it is essential that these studies focus on increasing animal productivity without forgetting animal health and sustainability in technological innovation processes.

### 3.6. Relevant Journal

The 187 sample articles on ammonia and GHG emissions from dairy cattle farming were published in 67 different periodicals, with ten of them responsible for 54.9% of the most relevant publications. Among these, five periodicals stood out, according to Table 1.

The five highlighted journals from 1987 to 2024 focus on the fields of dairy science, agriculture, engineering, and air quality.

The “Journal of Dairy Science” stands out as the most relevant journal due to the number of documents published (21). It serves as the official communication platform for the American Dairy Science Association. This journal focuses on publishing research related to the production and processing of milk or dairy products intended for human consumption. Consequently, it is a periodical reference for the academic and commercial dairy farming community.

“Biosystems Engineering” had the second-highest number of publications (15). This journal specializes in engineering and physical sciences publications applied to the sustainable development of land use and the environment.

The journals “Atmospheric Environment” and “Journal of Environmental Quality” primarily focus on gas emissions into the atmosphere. Despite ranking third in terms of the number of publications, “Atmospheric Environment” stands out with one of the highest citation counts for the period (68), demonstrating the value of these publications. As a result of these high metrics, this journal also achieved the highest H-index of the period, reflecting the balance between the number of publications and the number of citations [95]. Beyond its traditional use in evaluating individual scientists’ research output, the h-index is increasingly employed as a key metric for journals [96].

Ranked in fifth position is the journal “Animals”, with its first volume published in 2011. Its publications specialize in the study of animals, covering the areas of zoology, ethnozoology, animal sciences, animal ethics and animal welfare.

According to Table 1, the significance of interdisciplinary journals in scientific publications concerning emissions of gases from dairy facilities becomes evident. This characteristic suggests a strong interconnection between the specific research field and other disciplines, posing challenges in establishing a clear demarcation.

### 3.7. Most Relevant Papers and Their Characteristics

This section explores the most relevant articles on gas emissions in dairy cattle farming based on the number of citations. According to Table 2, it is possible to identify and analyze key works that have made significant contributions to the comprehension of gas emissions in dairy cattle. The number of citations, as shown in Table 2, ranks the ten most relevant articles.

Citations are used to measure the influence of an article; in other words, if an article is heavily cited, it can be considered important for the field of research [106]. All of these cited studies are focused on the topic of gas emissions associated with dairy production and animal waste management. The most cited papers explore several aspects, such as the impact of dairy cows’ diet on gas emissions, the technologies and management practices that influence these emissions, and the effects of feed additives on gas production. Additionally, there is research dedicated to the specific assessment of gas emissions such as ammonia, methane, carbon dioxide, and nitrous oxide, as well as studies on volatile organic compounds from dairy cows and their waste.

The most-cited study in the period analyzed was conducted by Amon et al. [97], who investigated the factors influencing the amount of CH_4_ and N_2_O and NH_3_ and means to reduce emissions. The authors examined all aspects of animal husbandry, from housing the animals in stalls to waste storage and distribution, under both aerobic and anaerobic conditions. They observed low emissions of NH_3_ and N_2_O within the animal pen. Moreover, they identified enteric fermentation as the primary source of CH_4_ emission. Regarding manure storage and distribution, NH_3_ levels increased by approximately a third after dispersion in the field, while CH_4_ and N_2_O emissions were higher in piled manure. The authors emphasized the importance of considering these harmful gases in waste management decisions, advocating for a comprehensive evaluation of the production system to mitigate emissions.

Gas emissions were also found to be related to different forage-to-concentrate ratios, as explored by Aguerre et al. [30]. This study, involving Holstein cows in a tie stall system, investigated CH_4_, CO_2_, and NH_3_ emissions resulting from various diets. While the proportions in the diet had no effect on NH_3_ and CO_2_ emissions, an increase in the forage-to-concentrate ratio led to elevated CH_4_ emissions, accompanied by increased fat content and decreased levels of protein, lactose, and solids in milk.

In terms of the addition of chemical additives to feed, Odongo et al. [102] demonstrated a reduction in CH_4_ emissions with the introduction of monensin. This substance not only decreased the percentage of fat and protein in milk but also did not adversely affect milk production or the animal’s dry matter consumption. The results suggest that incorporating this substance is a viable strategy for reducing CH_4_ production in lactating Holstein dairy cows. However, it is noteworthy to mention that monensin is among the frequently utilized ionophores in ruminant nutrition, despite the fact that ionophores are prohibited in some countries, such as the European Union [107].

### 3.8. Author Co-Citation Analysis

**Figure 7:**
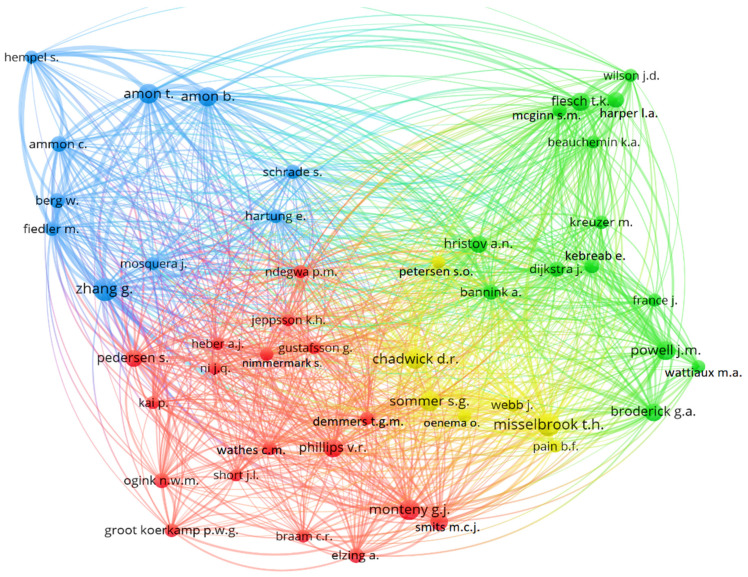
Scientific mapping of the co-citation of the most relevant authors on gas emissions in in dairy cattle systems.

By identifying the main authors with documents indexed in the Scopus and WoS databases, the relationships among them were obtained (Figure 7).

Co-citation analysis monitors pairs of papers cited together in the source articles. When the same pairs of papers are co-cited by many authors, clusters of research begin to form [108]. According to the same authors, the co-cited papers in these clusters tend to share some common theme. By employing techniques such as single-link clustering and multidimensional scaling, co-citation analysis has the capacity to map the structure of specialized research areas.

The author co-citation analysis depicted the network of author co-citation to illustrate the interrelationships among cited authors and identify those with significant influence [109]. To clearly demonstrate the author co-citation network, we only displayed authors with the most cited references. Only authors who had at least 30 citations were included in the selection process. This criterion allowed for the categorization of the 49 authors. They are grouped in clusters using k-means clustering.

In Figure 7, each node represents an author, with its size indicating the author’s impact, while the color denotes the field of knowledge (cluster) to which the study belongs.

The authors were grouped into four clusters identified by the color’s red, yellow, green, and blue, indicating similarities in their research lines. In the green category, researchers investigated GHG emissions from animal metabolism, primarily focusing on methane, ammonia and nitrous oxide [110,111,112]. Some of their works explore measurement techniques such as respiratory chambers, sulfur hexafluoride (SF6) tracer, and automated head chamber system, highlighting their effectiveness across various environmental conditions [113,114]. Furthermore, they emphasize the use of the reverse Lagrangian stochastic model (bLS) to establish the relationship between emission and concentration, using information such as wind speed and direction, surface roughness, and atmospheric stability [115].

The red category comprises authors who addressed gas emissions in dairy cattle facilities, mentioning gases such as carbon dioxide, methane, ammonia, and nitrous oxide [116,117,118]. These authors research gas emissions related to animal welfare, encompassing aspects such as management, nutrition, ventilation, atmospheric conditions influence, and measurement techniques [119,120,121,122].

The research conducted by the authors associated with blue is related to fluid dynamics and ventilation in the dispersion of gases, including carbon dioxide and ammonia [121,123]. They employ a variety of methods, including classical statistical modeling, machine learning, CO_2_ balance techniques, direct measurement of air flow and gas concentrations, as well as the use of non-dispersive infrared gas sensors (NDIR) [124,125,126,127,128]. Research focuses on environmental maintenance, energy consumption and production, and air quality.

Lastly, the yellow color indicates authors who focus their research on the sustainable management of agricultural waste, with an emphasis on ammonia and greenhouse gas emissions and evaluating the environmental impacts of intensive agriculture [129,130,131,132,133,134]. The research involves aspects of interaction between soil and atmosphere, including quantification, understanding processes, modelling, and development and evaluation of strategies to reduce emissions.

### 3.9. Relevant Institutions

The advancement of a field of knowledge can be achieved by identifying the institutions responsible for research. In bibliometric analysis, this information is fundamental for determining trends and relationships among these organizations [16].

**Figure 8:**
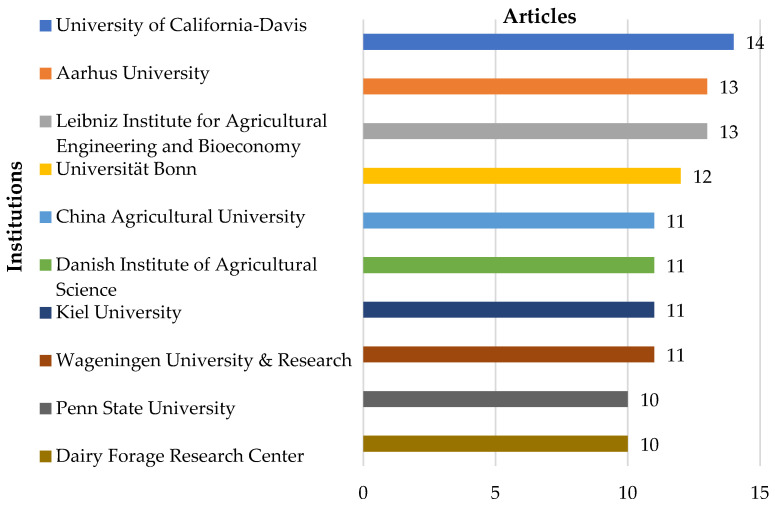
Most relevant institutions concerning gas emissions in milk production from 1987 to April 2024.

The ten most relevant institutions for the period from 1987 to April 2024 are represented in Figure 8.

The University of California–Davis has the highest number of publications (14) for the period. The State of California is a large producer of milk, which provides an incentive for researchers to investigate the environmental impacts caused by this sector [135,136].

Aarhus University in Denmark, appears with the second-highest number of publications (13), along with the Leibniz Institute for Agricultural Engineering & Bioeconomy in Germany. The University of Bonn, also located in Germany, follows closely with 12 publications. China Agricultural University, Danish Institute Athens, Kiel University, and Wageningen University & Research appear with the same number of publications (11).

Penn State University and Dairy Forage Research Center both have ten publications on GHG emissions in dairy production.

Institutions from Germany and the United States have published more of their research on GHG emissions. However, Europe has the largest number of institutions conducting research on the subject.

### 3.10. Keywords Related to NH_3_ and GHG Emissions in Dairy Cattle Housing Systems

**Figure 9:**
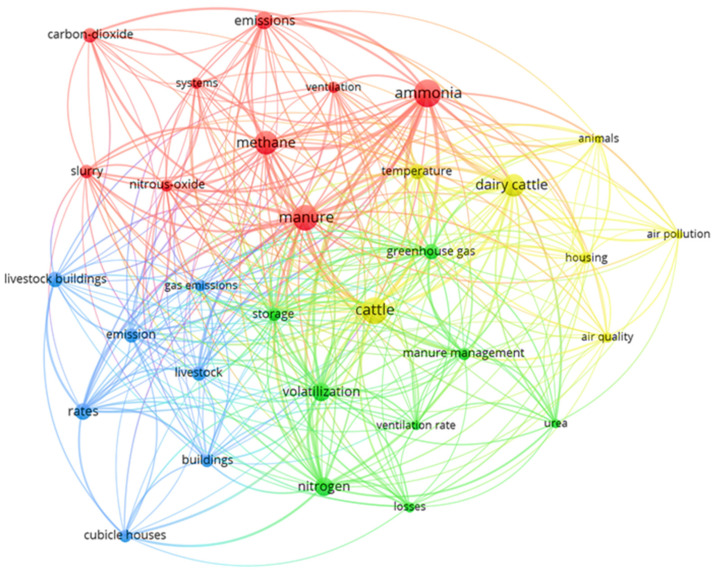
Map of network among author’s keywords. Lines indicate co-occurrences between terms. Red: the main studied gases. Yellow: related with the animal species and air quality. Green: manure management, nitrogen. Blue: livestock building. Lines indicate co-occurrences between terms.

One of the ways to present the results of the bibliometric analysis is to organize the most relevant authors’ keywords with the highest occurrence rates in the evaluated documents. The co-occurrence network offers a rapid method for visually comprehending the knowledge structure of literature on gas emissions in dairy cattle housing systems, utilizing authors’ keywords as variables [137]. Figure 9 illustrates the co-occurrence network and the most frequently utilized keywords in this field of research.

In Figure 9, circles can be identified where larger sizes indicate a higher occurrence of these keywords in the scientific articles studied regarding the emission of NH_3_ and GHG in dairy cattle facilities. Additionally, it can be observed that the main keywords are divided into four distinct clusters identified by the colors: yellow, red, blue, and green. The lines in the figure indicate the relationship between the keywords, showing which words co-occur in the articles.

In summary, the yellow cluster, centered around keywords like “cattle” and “dairy cattle”, sheds light on the primary focus of our evaluation—the animal species under scrutiny. Moreover, it reveals pertinent aspects concerning the environment and air quality within the housing system, exemplified by terms like “temperature” and “air quality”. As elucidated by Im et al. [138], temperature fluctuations hold the potential to significantly influence microbial activity and community dynamics within manure, consequently shaping the rates and overall magnitude of gas emissions.

The yellow cluster associates with the animal species we are evaluating, represented by words like “cattle” and “dairy cattle”, which are depicted by the largest circles. Additionally, it is possible to identify words related to the study of the environment and air quality inside the housing system, such as “temperature” and “air quality”.

The red cluster represents the main gases that appear in the evaluated research, highlighted by ammonia, methane, nitrous oxide, and carbon dioxide. The largest red circle emphasizes the keyword “manure”. It is known that many gases are emitted from livestock manure, and emissions of NH_3_, CH_4_, and N_2_O are important due to their indirect and direct effects on overall CO_2_-equivalent emissions [139]. These GHGs can be generated and released at every stage of the manure management continuum, including livestock housing, manure storage, treatment facilities, and land application of manure [140].

This topic is directly related to the green keyword cluster, which specifically addresses research related to nitrogen volatilization, manure management, and factors that may affect nitrogen volatilization emissivity. Dairy cattle manures contain substantial quantities of nitrogen, much of which is in inorganic forms [140].

Blue indicates keywords related to gas emission and livestock building. Among these, terms such as “cubic house” can be observed, highlighting key concepts in the study of environmental impacts and animal housing systems. Cubicle housing systems (cubicle barns), often called free stall barns, have been extensively utilized since the 1970s [77]. This housing system has been the most popular choice among dairy producers in recent years, leading to extensive research and consequently making it the most prevalent in scientific articles. This is the reason why this type of housing is the most prevalent in scientific research.

### 3.11. Trends in Gases Emission Research

**Figure 10:**
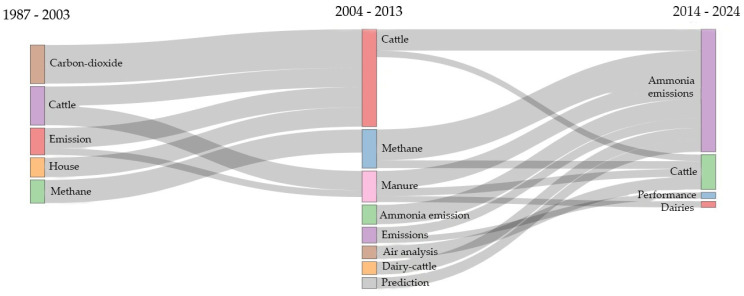
Main keywords expressed in years.

Figure 10 illustrates the primary keywords across three distinct periods within the studied years. The three-field plot serves as a dynamic tool for scientific and graphical mapping, summarizing the entire bibliometric study in a single figure and displaying proportionality among its contents [141].

It is observed that during the period of 1987–2003, the keyword that occurred most frequently was carbon dioxide. The emphasis on publications about carbon dioxide may reflect a greater recognition of the role of this gas in the context of climate change and global warming. Over the past 250 years, carbon dioxide has been considered the primary anthropogenic GHG [142], which may justify research on this gas.

Between the years 2004 and 2013, methane was the gas that had the highest appearance in scientific articles. The increasing concern with methane may be related to its significant contribution to the greenhouse effect and emissions from the livestock industry [143], especially intensive farming.

From 2014–2024, there was a noticeable exponential growth in scientific articles’ interest in conducting research related to ammonia. The exponential increase in interest in research related to ammonia since 2014 can be attributed to the growing recognition of the negative environmental impacts of this gas, especially concerning air quality, human health, and aquatic ecosystems. During the 20th century, there was a notable increase in research focused on ammonia, particularly after anthropogenic emissions were observed to exceed natural emissions for the first time [144]. This spurred efforts to devise solutions aimed at substantially reducing the emissions of this gas.

This trend suggests a change in the interests of researchers, policymakers, and society as a whole, as new information and environmental concerns emerge over time.

It is important to mention that with the development of new measurement technologies and their increasing popularity among researchers, new research emerges, and older studies may be updated. However, some gases, due to their specific characteristics, such as high volatility, require the development of more specific equipment and even more suitable physical structures to measure them. This makes it more difficult to conduct research, despite its recognized importance.

It is evident that these technologies require specialized researchers and significant financial investment, which limits many studies in various parts of the world. Additionally, there is political interest in these studies, which may finance research directed toward specific sectors that are of greater interest to them. Therefore, policymakers seeking to address some specific issue may turn to research for evidence-based solutions for making decisions on research directions and policy formulations [45].

The evolution in publications on gas emissions in dairy cattle systems has the potential to provide a basis for policies, steering sustainable agricultural approaches, and fostering public awareness regarding the impact of livestock agriculture on climate change, further emphasizing the dynamic nature of the field and its real-world implications.

### 3.12. Limitations of the Study

Several limitations were encountered during the course of this study. Firstly, it should be noted that the research was conducted exclusively in English, potentially excluding valuable insights from non-English sources, as mentioned by Silva et al. [17]. Additionally, many authors may benefit from including specific gas types or types of cow facilities in their titles, keywords, or abstracts to enhance visibility and relevance.

Furthermore, our evaluation was limited to peer-reviewed articles, thereby excluding potentially valuable contributions from book chapters, conference proceedings, and technical notes. While we focused on the top 10 most influential articles, recent publications may introduce paradigm shifts in the field [12]. However, it is important to acknowledge that these newer articles require time to accrue citations and garner attention.

Moreover, for a more comprehensive search, expanding beyond the Scopus and Web of Science databases to include additional research repositories could yield further insights. Another limitation to consider is the subjective nature of qualitative data. Researchers must exercise caution when drawing qualitative conclusions and ensure they are supplemented with appropriate analyses.

As highlighted by Wallin et al. [145], bibliometric analyses can only provide short-term forecasts of research trends, cautioning against overly ambitious projections of long-term impacts. Despite these limitations, a nuanced understanding of these constraints coupled with rigorous data standardization allows for the efficient interpretation of large datasets, thereby enhancing the research landscape.

## 4. Conclusions

Understanding ammonia and GHG emissions enables the development of more sustainable livestock production systems, aiming to minimize environmental impacts and climate change while improving animal welfare, productivity, and sustainability. Based on the results of this bibliometric analysis it is possible to highlight the growing importance of research on ammonia and GHG emissions in dairy housing systems and their influence on environmental sustainability and climate change. The paper offers an overview of the current state and emerging trends in scientific research and publications in this field. The bibliometric analysis explored in this paper provides valuable insights related to the evolution of scientific publications, research patterns, authorship trends, and citation networks regarding ammonia and GHG emissions in dairy housing systems. The results allowed us to identify the most relevant countries, journals, papers, institutions, researchers, and co-citation networks in the field. Additionally, it offers an evaluation of the main gases studied in the research and their importance.

From 1987 to 2024, there was a notable increase in scientific publications regarding ammonia and GHG emissions from dairy systems. These publications reflect a growing concern about gas emission sources, air quality, environmental sustainability, animal welfare, and gas mitigation strategies in dairy cattle housing systems.

According to the presented results, the main gases studied in the scientific articles were ranked in order of importance based on the quantity of articles published on the topic: ammonia, methane, carbon dioxide, nitrous oxide, and hydrogen sulfide, respectively. This order is attributed to the significance of these gases in animal comfort and well-being and also to their relevance concerning air quality and climate change. Understanding these gases is important not only for enhancing the living conditions of animals in livestock facilities but also for addressing broader environmental and global climate-related issues.

Most research/publications have been conducted in free stall facilities, probably due to their popularity among producers worldwide. The United States and Germany emerge as the leading countries in terms of the quantity of articles published on ammonia and GHG emissions in dairy facilities. The “Journal of Dairy Science” presents the highest number of publications (21), underscoring its relevance in disseminating research findings in this field.

This research can contribute not only to academic interests but also provide insights that enhance agricultural practices and inform the development of environmental policies. NH_3_ and GHG emissions in dairy cattle facilities are emerging as increasingly prominent and influential research fields, with implications extending from climate change mitigation to policy development for sustainable agriculture. Therefore, the relevance of this research is expected to persist over the years due to the importance of climate change and the increasing demand for sustainable agricultural practices. Moreover, there is a discernible trend towards expanding research in gas emissions and mitigation, with more researchers and organizations recognizing their importance and actively engaging in these areas. 

## Figures and Tables

**Table 1:** Most relevant journals in terms of relevant publications.

	Sources	SJR ^1^	Cite Score ^2^	JCR ^3^	H-i	ISSN	ND	NC
1°	Journal of Dairy Science	1.179	7.4	3.5	216	0022-0302	21	68
2°	Biosystems Engineering	1.061	10.1	5.1	125	1537-5110	15	13
3°	Atmospheric Environment	1.347	10.3	5.0	270	1352-2310	14	68
4°	Journal of Environmental Quality	0.803	6.6	2.4	183	0047-2425	14	15
5°	Animals	0.684	4.2	1.37	24	2076-2615	7	34

^1^ Scopus Index; ^2^ Scopus Index; ^3^ Web of Science Index; H-i: H Index; ND: Number of documents; NC: Number of citations.

**Table 2:** Journals with publications on gases emission.

R	Title	Authors	PY	Journal	NC
1°	Emissions of NH_3_, N_2_O and CH_4_ from dairy cows housed in a farmyard manure tying stall (housing, manure storage, manure spreading)	Amon et al. [97]	2001	Nutrient Cycling Agroecosystems	193
2°	Effect of forage to concentrate ratio in dairy cow diets on emission of methane, carbon dioxide and ammonia, lactation performance and manure excretion	Aguerre et al. [30]	2011	Journal of Dairy Science	164
3°	Ammonia emission from field applied manure and its reduction	Sommer et al. [98]	2001	European Journal Agronomy	155
4°	Emissions of ammonia, methane, carbon dioxide, and nitrous oxide from dairy cattle housing and manure management systems	Leytem et al. [99]	2011	Journal Environment Quality	141
5°	Emission of ammonia and other contaminant gases from naturally ventilated dairy cattle buildings	Zhang et al. [100]	2005	Biosystems Engineering	125
6°	Mitigation of greenhouse gas emissions in European conventional and organic dairy farming	Weiske et al. [101]	2006	Agriculture, Ecosystems & Environment	125
7°	Long-term effects of feeding monensin on methane production in lactating dairy cows	Odongo et al. [102]	2007	Journal of Dairy Science	119
8°	Greenhouse gas emission and nitrogen turnover in stored liquid manure nutrient cycling in agroecosystems	Sommer et al. [103]	2007	Nutrient Cycling Agroecosystems	115
9°	Greenhouse gas emissions from animal houses and manure stores	Jungbluth et al. [104]	2001	Nutrient Cycling Agroecosystems	108
10°	Volatile organic compound emissions from dairy cows and their waste as measured by proton-transfer-reaction mass spectrometry	Shaw et al. [105]	2007	Environmental Science & Technology	96

R: Ranking; PY: Publication Year and NC: Number of Citations.

## Data Availability

Not applicable.
